# Supplementary material for: Three decades of ethical, legal, and social implications research: Looking back to chart a path forward
Source: Cell Genom. 2022 Jun 27;2(7):100150. doi: 10.1016/j.xgen.2022.100150 (PMC9352173; doi:10.1016/j.xgen.2022.100150)
Supplement: Document S2. Article plus supplemental information [file mmc2.pdf]

Cell Genomics

CellPress
OPEN ACCESS

Perspective

Three decades of ethical, legal, and social implications research: Looking back to chart a path forward

Deanne Dunbar Dolan,[1,*] Sandra Soo-Jin Lee,[2] and Mildred K. Cho[3]
[1]Center for ELSI Resources and Analysis (CERA), Stanford Center for Biomedical Ethics, Stanford University School of Medicine, Palo Alto, CA 94305, USA
[2]Division of Ethics, Department of Medical Humanities & Ethics, Columbia University, New York, NY 10032, USA
[3]Departments of Medicine and Pediatrics, Stanford Center for Biomedical Ethics, Stanford University School of Medicine, Palo Alto, CA 94305, USA
*Correspondence: ddolan@stanford.edu
https://doi.org/10.1016/j.xgen.2022.100150

SUMMARY

More than thirty years ago in the United States, the National Center for Human Genome Research (NCHGR) at the National Institutes of Health (NIH) and its partner in the Human Genome Project (HGP), the Department of Energy (DOE), called for proposals from social scientists, ethicists, lawyers, and others to explore the ethical, legal, and social implications (ELSI) of mapping and sequencing the human genome. Today, nearly twenty years after the completion of the HGP, the ELSI Research Program of the National Human Genome Research Institute (NHGRI) continues this support. It has fostered the growth of ELSI research into a global field of study, uniquely positioned at the nexus of many academic disciplines and in proximity to basic and applied scientific research. We examine the formation of the first ELSI program and consider whether science policy in the public interest can exist within the confines of a set-aside from the NHGRI budget.

INTRODUCTION

In the U.S., "ELSI" refers to the field of study concerned with the ethical, legal, and social implications of genetics and genomics (Table 1). Between its origins in the international Human Genome Project (HGP) (which sequenced and mapped the complete human genome from 1990 to 2003) and today, ELSI scholars have produced thousands of articles, books, and other materials.[1] These works explore a variety of issues in basic research and its clinical translation, as well as broader societal issues raised by emerging technologies in the life sciences.[2] Globally, ELSI researchers are positioned in proximity to, or embedded in, large life science initiatives; focus on the anticipation of, or rapid response to, emerging scientific issues; support the co-design of research agendas with the public; interact with a broad range of stakeholders (the media, policy makers, and industry); and use diverse source materials and approaches (see Table 2).[3,4] Although heterogeneous in its methods, it is our contention that ELSI inquiry is focused on distinct objects for unique purposes compared with adjacent fields, such as bioethics, and nearby disciplines.

ELSI researchers identify and explore issues associated with the conduct and application of scientific research.[15] There are many reasons to take up this study, including facilitating public discussion and ensuring that these explorations are conducted for the benefit of society, their benefits are evenly distributed, and harms and misuse are limited to the greatest extent possible.[21] These critical missions are not necessarily oppositional to scientific discovery. As their work is in the public interest, ELSI researchers strive for intellectual independence from both the science they observe and institutional or financial pressures that conflict with their mission. We proceed from the premise that although smaller sources have funded U.S. ELSI work and the "ELSI community" is enriched by scholars without grant-based funding, substantial and sustained funding by the ELSI Research Program at the National Human Genome Research Institute (NHGRI) has been, and remains, a primary support to the field of study.[1,22–24] In this paper we consider "the ELSI hypothesis"—the idea that the best way to produce informed science policy for the HGP would be a formal budget "set-aside" within it—and offer some lessons to inform the future direction of the ongoing U.S. federal funding program.

ESTABLISHING ELSI RESEARCH IN THE UNITED STATES

In 1990, the international effort to map and sequence the human genome promised to dramatically enhance our understanding of human biology and of both genetic and acquired diseases.[25] However, in light of atrocities inspired by eugenics movements, which in America had provided the rationale for state-sponsored mass sterilization programs and in Europe had inspired the Holocaust perpetrated by Nazi Germany, it was clear that the ability to ascertain genetic information would bring with it the possibility

This is an open access article under the CC BY license (http://creativecommons.org/licenses/by/4.0/).

Table 1. Acronym definitions

BERAC	Biological and Environmental Research Advisory Committee (Department of Energy)
CEER	Center of Excellence in ELSI Research
CERA	Center for ELSI Resources and Analysis
DHHS	Department of Health and Human Services
DOE	Department of Energy
ELSI	Ethical, legal, and social implications
ERA	ELSI Research Advisors
ERPEG	ELSI Research Planning and Evaluation Group
GSWG	Genomics and Society Working Group
HGP	Human Genome Project
NACHGR	National Advisory Council for Human Genome Research
NIH	National Institutes of Health
NCHGR	National Center for Human Genome Research
NHGRI	National Human Genome Research Institute (formerly NCHGR)
OHER	Office of Health and Environmental Research, Office of Energy Research, Department of Energy
OPCE	Office of Policy, Communications, and Education (NIH)
RAC	Recombinant DNA Advisory Committee

of stigma and discrimination for carriers of genetic disorders, inhibit equitable access to U.S. health care, and even change the composition of human society, if genetic information were used to shape reproductive planning.[26–29] The architects of the HGP could not ignore the cultural currents of the preceding decades, including the nature or nurture debates in psychology, the rise of global human rights discourses, or the evolution of medical ethics and human subjects protections.[30] In fact, both the National Academy of Sciences and the Congressional Office of Technology Assessment had discussed ethical and social issues in the planning and feasibility reports for the HGP.[31,32]

Speaking to these issues, James Watson, co-discoverer of the molecular structure of DNA and the first HGP director at the National Institutes of Health (NIH), announced at an October 1988 press conference that a portion of the project budget would be used to study its impacts on society.[33,34] The first action in this direction was the establishment of a working group on ethics in January 1989 to coordinate efforts at the NIH and the Department of Energy (DOE), the two primary U.S. HGP agencies (for the DOE interest, see Annas and Elias[35]).[36] In March 1989, the Office of Human Genome Research (OHGR) added a program announcement to the NIH Guide to Grants and Contracts to request the first applications for ELSI research (see Box 1).[36]

The working group on ethics held its first formal meeting on September 14 and 15, 1989, one month before the Department of Health and Human Services (DHHS) established the National Center for Human Genome Research (NCHGR) at the NIH.[36,37] This meeting resulted in a mission statement that inspired the NCHGR to establish an ELSI branch within its Division of Extramural Research and the DOE to start an ELSI program in the Office of Health and Environmental Research (OHER) and a revised NIH program announcement.[36,38] ELSI grant applications from members of the academic research community were now encouraged in nine topic areas (see Box 2). The parallel DOE grants program was preferentially focused on privacy, fair use of genetic information, and education of the public.[33,36,39]

In November 1990, the two agencies announced the formation of the Joint Working Group on Ethical, Legal, and Social Issues (the "ELSI working group") to steer the course of the two extramural research programs, convene various task forces and conferences, and coordinate the production of policy options.[39,40] It was initially chaired by Nancy S. Wexler, PhD, a clinical psychologist, and comprised experts in law, ethics, genetics, clinical medicine, and other fields.[41] During its tenure, the group established task forces on privacy and insurance and directed the distribution of grant funds for research on access to high-quality genetic tests, the fair use of genetic information by employers and insurers, privacy issues, and public and professional education.[29,42,43] By September 1991, the NCHGR had funded twenty-five extramural ELSI grants and ten national conferences, including a January 1991 workshop that resulted in a social policy research agenda that is of continued relevance today (see Box 3).[35,39,40]

ELSI research grants received 3% of the annual budget of the NCHGR, with a budgeted scale up to 5% within the first three years (this occurred in 1991) and a consistent 3% of the OHER/DOE budget.[33,34,43] In addition to the funds that were earmarked for ELSI research grants, the activities of the ELSI working group were jointly funded from the administrative budgets of the NCHGR and DOE.[44] The financial commitment by the DOE was the idea of Tennessee Democratic senator Al Gore, who challenged a DOE official in a 1989 subcommittee hearing, upon learning that the agency had not created an ELSI set-aside: "I think that whether you set aside the money or not will be a signal of whether you are really taken seriously or not. I would like to see not so much a duplicate of the NIH effort as I would like to see in the joint plan you develop a comparable commitment of money."[45] The total sum of this first-of-its-kind ethics

CellPress
OPEN ACCESS

Table 2. Examples of global ELSI initiatives, 1990 to present

Location	Date	Initiative(s)
Austria, Finland, and Germany	2009	Austria, Finland, and Germany launched a multinational initiative called ELSAGEN to fund collaborative research on ELSA issues associated with genomics and the related sciences.[5,6]
Canada	2000 to present	Genome Canada, a not-for-profit corporation partly funded by the Federal Government of Canada, funds the Genomics, Economic, Ethical, Environmental, Legal and Social Aspects (GE^{3}LS) program.[5,7]
	2001–2011	The Canadian Institutes of Health Research, Institute of Genetics, includes the study of genetics and the ethical, legal, and social issues it raises as a strategic research priority.[5]
	1992–1997	The Medical, Ethical, Legal and Social Implications (MELSI) of genetics program was a component of the Canadian Genome Analysis and Technology (CGAT) initiative, the Canadian contribution to the HGP.[7,8]
European Union	2013–2020	The European Commission made responsible research and innovation (RRI) a cross-cutting theme in the Framework Program for Research and Innovation, Horizon 2020, and assigned responsibility for RRI to the Science with and for Society (SwafS) sub-program. RRI themes include public engagement, open access, gender, ethics, and science education.[9,10]
	2002–2012	The Economic and Social Research Council funded centers and institutions across the United Kingdom (Cesagen, Innogen, Egenis, and Genomics Forum) to study the economic and social implications of genomic science and technologies. Together, these centers were called the Economic and Social Research Council (ESRC) Genomics Network or EGN.[5,11]
	1994–1998	The 4th European Union Framework Program introduced ELSA as a label for funding research into the ethical, legal, and social aspects of emerging sciences and technologies, stakeholder dialogues, education, and other activities.[3,12]
The Netherlands	No date available	The Societal Component of Genomics (MCG) program of the Dutch Research Council or Netherlands Organization for Scientific Research (NWO) funded researchers in the social sciences and humanities to anticipate developments in science and society.[6,3,13]
	2009–2011	The Dutch government organized the Committee Societal Dialogue Nanotechnology (CieMDN) and tasked it with organizing a national public dialogue on nanotechnology called Dutch Nanodialogue that was active from March 2009 to January 2011.[14]
	2005–2010	The ELSA (ethical, legal, and societal aspects) coordinating project, Societal Aspects of Genomics of the Sixth Framework Program project, ERA-SAGE, was coordinated by the Netherlands Organization for Scientific Research beginning in 2005. It coordinated ELSA activities in eight national funding agencies (the Netherlands, United Kingdom, Austria, Norway, Finland, Germany [2], and Canada) and three funding agencies with a specific interest in this field (Israel [2] and Switzerland). It has been argued that ELSA in these countries expanded beyond genomics in 2005 and began to be applied to other emerging technologies, such as nanotechnology, information and communication technologies, synthetic biology, and neurotechnology.[15,16]
	2004	The Netherlands Genomics Initiative (NGI) allocated 5% of its budget to two initiatives: (1) researcher-driven projects on “the societal component of genomics research” and (2) the Centre for Society and Genomics (CSG) (later renamed the Centre for the Study of Life Sciences), which housed ~50 ELSA research projects.[3,4,5,6]
	2001	The Dutch government allocated €189 million to genomics research and earmarked 4% for the study of ethical, social, economic, psychological, and legal aspects of the genomics programs and the establishment of the Netherlands Genomics Initiative (Nationaal Regie-Orgaan Genomics), an independent task force charged with governing the new genomics infrastructure.[17]

(Continued on next page)

Table 2. *Continued*

Location	Date	Initiative(s)
Norway	2014 to present	In addition to the Research Council of Norway (RCN) ELSA I and ELSA II programs, early Norwegian national biotechnology programs, such as those for functional genomics (FUGE) and nano materials (NANOMAT), include ELSA research components. Ongoing programs, such as BIOTEK2021 and NANO2021, allocate 2%–5% of their funding to ELSA.[18]
	2008–2014	The ELSA II program period was focused on nanotechnology and new materials.[15,18]
	2002–2007	The ELSA of Nanotechnology, Biotechnology and Neurotechnology Program was established by the Research Council of Norway (RCN) to study issues associated with biotechnology, nanotechnology, and cognitive science. The first program period (ELSA 1) focused on functional genomics.[5,6,15,18]
South Korea	2001	The South Korean government funded an ethical, legal, and social implications program.[6,19,20]

set-aside within a major U.S. scientific initiative inspired Dr. Wexler to describe the ELSI program as the "the largest biomedical ethics program in this country and probably in the world."[34,46,47]

In June 1993, Congress formally instantiated the activities of the ELSI branch at the NIH with legislation called the National Institutes of Health Revitalization Act. This act mandated that "not less than 5 percent" of the NCHGR budget be allocated to "reviewing and funding proposals to address the ethical and legal issues associated with the genome project (including legal issues regarding patents)."[48] That same year, Francis Collins succeeded Watson as director of the NCHGR, with oversight of the HGP and the ELSI branch. He added two professional staff positions and expanded the group to include representation from lay constituencies, clinical professions, and genome science.[33] In 1995, at the end of Dr. Wexler's 5-year term as chair of the ELSI working group, Lori Andrews, a law professor and legal scholar, was elected to replace her.[49]

RETHINKING ELSI AT THE NATIONAL INSTITUTES OF HEALTH

Early in 1996, an internal dispute about the purpose and autonomy of the ELSI programs arose when Collins vetoed a plan for an anthology on behavioral genetics. Citing interference by the NCHGR in the budget allocation and position statements of the ELSI working group, Andrews resigned in protest in February 1996. Sociologist Troy Duster, who temporarily replaced her, urged that the working group be made autonomous from NCHGR and highlighted the imbalance between allocations to genome science and ELSI.[12,34,44,49,50] In a context in which the "growing gap between diagnostic information and therapeutic capacity is a time-bomb," Duster opined, "the formula for 95% for the mapping and sequencing versus the 5% for the social consequences seems particularly absurd. What about 50:50?"[44]

Reflecting on her experience, Andrews expressed concerns that Collins had "begun to stack" the ELSI working group by adding a genome scientist as a voting member without nomination or vote by the group.[49] Collins denied that he prevented the working group from expressing opinions but conceded the necessity of "some limits to its autonomy because it is not a free-standing commission."[44] Following her resignation (and perhaps, as Andrews said, as an investigation into her resignation), Collins and Dr. Ari Patrinos, associate director for health and environmental research at the DOE, commissioned an independent review to evaluate the scope of ELSI activities, the role of external advisers in the ELSI program, and how best to structure input on ELSI issues. They appointed the Committee to Evaluate the Ethical, Legal and Social Implications Program of the Human Genome Project (ELSI evaluation committee) on April 30, 1996.[44,49,51]

The December 1996 report of the ELSI evaluation committee found the ELSI working group to be an integral part of the HGP with a mandate "too broad to be satisfied by any single body" and placement "not commensurate with the more global role of some important policy formulation." The report offered three recommendations: (1) that the NIH implement a process for communication and coordination of the ELSI activities on research ethics in genetic studies within the institutes; (2) that it restructure the existing working group into the ELSI Research Evaluation Committee, which would coordinate ELSI grants and set the research agenda; and (3) that it establish a federally chartered advisory committee on genetics and public policy situated in the Office of the Secretary of the Department of Health and Human Services to "assume the role of identifying issues and formulating policy to ensure integration of new genetic knowledge into health care standards."[51] The remainder of the ELSI working group resigned following the external evaluation, in expectation of the proposed new configuration.[49] Thus, it had ended its advisory role to the NHGRI and DOE ELSI grant-making programs, its task force work, and coordination of policy options by 1997.[52]

Also in 1997, the DHHS elevated the NCHGR to the status of a research institute and renamed it NHGRI.[53] At its February 1997 meeting, the National Advisory Council for Human Genome Research (NACHGR) endorsed all three ELSI evaluation committee recommendations. In July 1997, the NACHGR and the Biological and Environmental Research Advisory Committee (BERAC) at the DOE formally established the ELSI Research

Box 1. Applicants were asked to address the following questions about research on the human genome in the NIH Guide to Grants and Contracts, 1989

- What are the concerns to society and to individuals?
- What questions in the areas of ethics and law need to be addressed?
- What can be learned from precedents?
- What are the policy alternatives and the pros and cons of each?
- How can we inform and involve the public?[36]

Planning and Evaluation Group (ERPEG). The ERPEG, chaired by ethicist LeRoy Walters, analyzed the portfolio of ELSI research grants, engaged in a strategic planning process that resulted in the ELSI component of the 1998–2003 HGP strategic plan, and provided expert guidance on both extramural ELSI research portfolios until January 2000.[38]

The ERPEG presented its final report at the February 27, 2000 NACHGR meeting. It noted that the NHGRI had spent more than $58 million on the ELSI program and that the DOE had spent $18.2 million at the end of fiscal year (FY) 1999. The committee recommended that the DOE expand the staffing of its program beyond a single individual, a new joint planning and evaluation group, the recruitment of investigators from underrepresented groups, and activities to promote collaboration between HGP scientists and ELSI researchers, citing the existence of "some in the scientific community who remain indifferent or even hostile to ELSI research."[52] Their portfolio analysis noted that the R01 mechanism may account for the very small number of legal, philosophical, theological, sociological, or economic analyses. They noted highly cited publications, policy recommendations (especially a draft "genetic privacy act" by George Annas of Boston University), and the formation of two successful research consortia, among other program accomplishments.[52,54]

With guidance from the ERPEG, Collins and colleagues identified goals for the ELSI Research Program in the HGP strategic plan for 1998–2003, focused on anticipating the reception and use of the results of the project in clinical care, prevention, nonclinical, and policy settings (see Box 4).[55] The ERPEG recommendation to re-establish a joint DOE/NHGRI ELSI planning group did not come to fruition. However, in 2000, NHGRI formed the ELSI Research Advisors (ERA), a subcommittee of the NACHGR, to advise the council on the grants program and plan for the role of ELSI following the completion of the genome sequencing work.[56] ERA produced a white paper in 2003 that called for enhanced integration between the ELSI extramural grants program and the Office of Policy, Communications, and Education (OPCE) within the NHGRI Office of the Director, among other recommendations. The white paper identified the NHGRI as a "federal agency responsible for funding research" with "no mandate to oversee the development of policies" and suggested a division of labor in which ELSI researchers "develop a body of knowledge" and the OPCE translate their findings to policy makers.[56,57]

In its 2005 report to the NACHGR, the ERA identified three "persistent challenges" that should be addressed for the ELSI Research Program to fulfill its mission: (1) increased integration between ELSI and genome research, (2) more effective translation of ELSI research findings into products that can inform policy, and (3) expansion of the disciplinary and demographic diversity of the ELSI community of researchers. Echoing its white paper and a 1992 report by the House Committee on Government Operations, the report had this to say: "as an extramural research program housed within the Federal Government, ELSI is statutorily not capable of developing or presenting in an effective manner specific policy recommendations to the Nation, the Congress, or the executive branch on the full range of problems presented by the Human Genome Project."[43,57] However, the ERA report implied that the translation of academic research to policy was within the mission of the ELSI Research Program because, among others who might do this work, its grantees were most conversant with ELSI research findings. The report offered that the range of expertise in the ELSI research community should continue to expand and noted that the Centers of Excellence in ELSI Research (CEER) consortium funded beginning in September 2004, with a goal of "translat[ing] ELSI research to safe, effective, and just genetic and genomic policies and practices in research, health, and non-medical settings" would partly address translation challenges.[57]

RECENT NHGRI ELSI FUNDING PRIORITIES

Like its prior strategic plan, the NHGRI objectives for 2011–2021 emphasized the continued translation of genetic science into the clinical setting, including training clinicians to interpret and use genomic data.[58] The DOE ultimately retired its smaller ELSI grant program. Its NHGRI counterpart continued. The 2012 NHGRI reorganization by its director, Eric Green, located the ELSI extramural research program within the Division of Genomics and Society.[59] Today, planning and priority setting at the division

Box 2. Research topics in the 1990 revision of the NIH program announcement for those seeking grants to conduct ELSI research

1. Fairness in insurance, employment, the criminal justice system, education, adoption, the military, and other areas
2. Psychological and societal responses to individual genetic information
3. Privacy and confidentiality, including ownership, control, and consent
4. Genetic counseling in conjunction with testing for prenatal, pre-symptomatic, and polygenic conditions and carrier status; testing in the absence of therapeutic options; and population screening versus testing
5. Genetic information and reproductive decisions
6. Introduction of genetics into medical practice, including standards of care, qualification of medical professionals, and education of patients and the public
7. Historical misuses of genetics, especially eugenics, and relevance to the present
8. Commercialization, including property and intellectual property rights, and accessibility of data and materials
9. Philosophical issues such as definitions of health and disease and questions of determinism and reductionism[36,40]

Box 3. Social policy research priorities for the Human Genome Project, 1991

1. When and how should new genetic tests be introduced into medical practice?
2. How can the confidentiality and privacy of an individual's genetic information be preserved?
3. How can genetic discrimination by employers and insurance companies be prevented?
4. How might the Human Genome Project affect our concepts of "disease," "normalcy," and "humanness"?

Source: ELSI Priority-Setting Workshop (January 1991), National Center for Human Genome Research, National Institutes of Health, Bethesda, Maryland.[35]

are directed by the NACHGR, with advice from the Genomics and Society Working Group (GSWG) and periodic strategic planning processes.[38,60] NHGRI still allocates at least 5% of its annual extramural research budget to the ELSI Research Program (see Table 3). This amounted to $1.57 million in fiscal year 1990, $18.9 million in 2016, and about $22 million in 2020.[60,61] In 2020, program funds were allocated between investigator-initiated research (68% of $22 million) and program-initiated research (32%) (J. Boyer, personal communication). This sustained level of financial commitment has made the field of genomics unique among U.S. biosciences.[2]

Today, the ELSI Research Program, often in partnership with other NIH institutes or centers, funds U.S. investigators in four overlapping research areas: genomics and sociocultural structures and values, genomics at the institutional and system level, genomic research design and implementation, and genomic health care.[62] In the training category, the program provides institutional research training grants (T32) to support three pre- and post-doctoral training programs in ELSI research, among other support to trainees and early-career investigators.[60] In fiscal year 2020, 18% of the ELSI budget (about $4 million), was spent on the various training programs (J. Boyer, personal communication).

The ELSI Research Program has also invested in activities to build institutional capacity for ELSI research, translate ELSI research, and encourage collaboration among the ELSI scholarly community. For example, in 2004, NHGRI collaborated with the DOE and the Eunice Kennedy Shriver National Institute of Child Health and Human Development (NICHD) to establish the first four CEERs.[61,63] In 2020, it continued the funding for three CEERs (see Table 4) using a limited competition request for proposals (renewal of current CEERs only) with the indication that "NHGRI plans to maintain the CEER program at approximately its current level of funding through FY 2023."[64] In 2019, it funded the Center for ELSI Resources and Analysis (CERA) to build the community ELSI researchers and provide a web-based platform to enhance the production, sharing, and use of ELSI research (for CERA rationale, see Oliver and McGuire,[65] Kaye et al.,[66] and Bell et al.[67]). Although a small portion of the budget, the program has also provided formal support for ELSI studies embedded in large genomics initiatives sponsored by other NHGRI divisions such as the CSER Consortium, eMERGE, the Human Microbiome Project, the Wellcome Trust/NIH H3Africa Initiative, and the NBSeq initiative, as well as supplements to other NIH grants with ELSI components.[60,61,63,68–70]

ELSI CRITICISMS AND IMPACTS

Over the past 30 years, criticisms have come from scientists, NIH officials, and ELSI researchers themselves. Early on, HGP scientists questioned whether funding the study of consequences was a good use of project funds and worried that an ethics component would signal a need for public scrutiny.[12,40] Because ELSI research shares funding with the science it observes, other commentators have said that ELSI researchers have no choice but to function as translators, mediators, or facilitators of science by manufacturing public acceptability.[71,72] Andrews reported that Watson made a remark at a genetics policy meeting implying that he had configured the ELSI program to minimize its ability to impede HGP progress: "I wanted a group that would talk and talk and never get anything done and if they did do something, I wanted them to get it wrong. I wanted as its head Shirley Temple Black."[49,73] Internal participants and external observers alike viewed the diversion of HGP funds to ELSI as an "unavoidable political tax" that Watson was willing to pay to accomplish his scientific goals.[29] For their part, bioethics scholars worried that ELSI funding would direct the attention of social scientists and humanists away from other pressing issues in biomedicine while at the same time reducing the capacity of bioethicists to critically examine the HGP "either by professionally indebting them to the Project or by redirecting their attention 'downstream' from the Project to its applications."[33]

Scholars in the ELSI community have identified epistemological differences between ELSI researchers, scientists, and clinicians; time pressure; knowledge gaps on both sides; and power imbalances in collaborative relationships as barriers to effective, real-time consideration of ELSI issues.[71,74,75] For thirty years, there has been active discussion over whether ELSI research has had or should have a direct policy impact.[3,29,40,63,76,77] Another criticism is that the focus by ELSI researchers on the "implications of" novel technologies requires its practitioners

Box 4. Goals for the ELSI Research Program in the HGP strategic plan for 1998–2003

- Examine the issues surrounding the completion of the human DNA sequence and the study of human genetic variation.
- Examine issues raised by the integration of genetic technologies and information into health care and public health activities.
- Examine issues raised by the integration of knowledge about genomics and gene-environment interactions into nonclinical settings.
- Explore ways in which new genetic knowledge may interact with a variety of philosophical, theological, and ethical perspectives.
- Explore how socioeconomic factors and concepts of race and ethnicity influence the use, understanding, and interpretation of genetic information, the use of genetic services, and the development of policy.[25]

Table 3. Summary of the National Human Genome Research Institute extramural research budget, 2018–2021

Year	2018	2019	2020	2021
NHGRI extramural research budget	$391,000,000	$405,000,000	$430,000,000	$437,000,000
ELSI budget	$21,000,000	$21,000,000	$22,000,000	$23,000,000
Percentage of the NHGRI extramural research budget allocated to ELSI	5.37%	5.19%	5.12%	5.26%

to use "speculative ethics" and make policy recommendations on the basis of "a possible (and probably inadvertent) exaggerated portrayal of harm" instead of evidence. These commentators raise concern that ELSI findings are conveyed without appropriate nuance and that the resulting "ethics hype" can misinform the public, lead to poor policy decisions, and create backlash against promising research fields.[71,72,78,79]

Despite these criticisms, commentators have credited ELSI research programs throughout the world with the creation of "a healthy culture of skeptical scrutiny" useful for the examination of emerging science and technology.[61,78] More tangibly, ELSI research has been an upstream contributor to several important legislative and judicial outcomes. For example, in 1992, the recommendations of the ELSI Task Force on Genetic Information and Insurance were passed on to the White House Task Force on Health Care Reform, chaired by Hillary Clinton. These recommendations were included in the Health Care Security Act of 1993 and became the public case for health care reform.[33,40] Although the bill failed, the Health Insurance Portability and Accountability Act of 1996 ultimately excluded indications for developing genetic disease as predicted by genetic tests (in the absence of disease) from the list of clauses naming preexisting conditions.[40] ELSI has also been credited with influencing the Congress to extend the Americans With Disabilities Act to offer protection from employment discrimination to individuals with genetic disease or test results predicting the clinical manifestation of future genetic disorders.[40,80] ELSI research findings informed a report by the NIH Secretary's Advisory Committee on Genomics, Health, and Society (SACGHS) that became part of the evidence for the Supreme Court finding against Myriad Genetics, which argued that DNA was excluded from patent eligibility. This important decision contradicted the then generally accepted practice of gene patenting.[61]

ELSI research has also produced several concrete policy outcomes that have positively shaped the conduct of genomic research and protected human rights. Among these are the Genetic Information Nondiscrimination Act of 2008 and the Universal Declaration on the Human Genome and Human Rights.[78,81–84] Other important ELSI accomplishments are improvements to the drafting and ethical review of consent forms for genomic studies; the development of NIH policies for genomic data sharing for the purposes of conducting genome-wide association studies (GWAS); position statements, policies, and recommendations for direct-to-consumer genetic testing; policies, practices, and governance for biobanks and biorepositories at the NIH and other institutions; the adoption of genetic screening guidelines by professional organizations; an executive order protecting federal employees from genetic discrimination in the workplace; analysis and recommendations on returning individual results to research participants; and recommendations to end the clinical and research use of race as a biological category.[53,61,78,85–91]

DISCUSSION

One interpretation of the institutional history of the ELSI program is that the 1996 evaluation ordered by Collins and Patrinos was a deliberate strategy designed to end the ELSI working group, motivated by its ability to set an agenda that was not in alignment with NIH priorities. It could be argued that the group had a direct role in policy formulation because it could operate like an independent commission (as evinced by its task forces on insurance and privacy and draft genetic privacy legislation). Compared with the ELSI working group, the subsequent ELSI advisory groups formulated by NHGRI in its wake, for example, ERPEG (1997–2000), ERA (2000–2012), and GSWG (2012 to the present), could be seen to have a reduced and more internal influence on the NIH scope of work, namely, to analyze the portfolio of ELSI grants and suggest priority areas to the NACHG. Whether this move by Collins was, as Andrews suggested in 1999, an attempt to curtail the "independence" of the ELSI working group is for the reader to decide.[49]

An alternative interpretation is that the 1996 evaluation, even if it had ended the ELSI working group, did not end the production of ELSI-informed science policy. Instead, it catalyzed the transfer of its policy functions to other entities such as the Trans-NIH Bioethics Committee (created in July 1997 by Collins); the Secretary's Advisory Committee on Genetic Testing (SACGT) (chartered by the secretary of health and human services in June 1998), and its successor through 2011, the Secretary's Advisory Committee on Genetics, Health, and Society (SACGHS); and the National Bioethics Advisory Committee (1996–2001), and its successor the President's Bioethics Commission (2001–2009).[57] Although some of these entities were not specifically concerned with genetics policy, SACGHS, which was situated within the NIH Office of Science Policy and was charted to formulate recommendations for the DHHS and other federal agencies on "the range of complex and sensitive medical, ethical, legal, and social issues raised by new technological developments in human genetics," had a remit that closely paralleled the aims of the ELSI working group.[92] In addition, other NIH committees such as the Recombinant DNA Advisory Committee (RAC) (and its successor NexTRAC in the NIH Office of Science Policy) have advised the NIH Office of the Director on ethical, legal, and social issues of emerging technologies beyond genetics. However, few of these entities can be characterized as truly independent of NIH priorities.

Before the ELSI working group and the ELSI grants programs at the NIH and the DOE, ethical oversight of biomedical research in the United States was the remit of independent commissions

Table 4. Centers of Excellence in ELSI Research funded by the National Human Genome Research Institute through 2024

Center of excellence	Focus area(s)	Institution
Center for the Ethics of Indigenous Genomic Research	Research, education, and outreach for ethical genomic research in partnership with American Indian and Alaska Native communities	University of Oklahoma
Genetic Privacy and Identity in Community Settings (GetPreCiSe)	ELSI issues involving genetics, privacy, and identity; related laws and regulatory frameworks; privacy protection technologies	Vanderbilt University Medical Center
University of Utah Center of Excellence in ELSI Research (UCEER)	ELSI issues in population-based genetic testing and screening (e.g., newborn screening, prenatal screening, carrier screening, etc.)	University of Utah

or agency-based advisory panels. These bodies would recommend studies that would then be funded by targeted government contracts or grants.[93] In the initial years of the ELSI extramural grants programs, at least one science policy scholar suggested that a federally chartered commission would have been superior to the ELSI model because the research programs (1) excluded all citizens from participation in setting the policy agenda, except those capable of responding to a grant solicitation, and (2) academicians are "dangerously naive when it comes to public policy."[29] A 1992 report by the House Committee on Government Operations recommended legislation to establish an advisory commission on the ethical, legal, and social implications of the human genome project supported with a portion of the ELSI grant funds because the NIH-DOE ELSI programs are principally designed to support academic research."[43] However, the superiority of commissions (whether NIH dependent or independent) in comparison with the ELSI model did not have universal support.[33,93] Although concerns about the intellectual independence of the field are still present in the minds of ELSI scholars and others today, the decision to establish the HGP as the first federally funded, scientific research program to allocate a portion of its funding to the study of its own impact has been called a "disruptive leap."[93] The "un-commission" (as Eric Juengst described the ELSI Research Program) can be seen as a competitively funded, investigator-initiated research program open to all U.S. scholars, that ensures oversight is near scientific developments on an ongoing basis.[93]

It is our view that the public interest is best served if national science policy is formulated by agencies external to the NIH. However, before the ELSI Research Program at NHGRI, there was virtually no literature (except for narrowly focused technology assessments and sociological studies of technological process) with which to think about the social impacts of a new technology and what action to take. Our interpretation of the ELSI experiment is that to maximize the public benefit of such research, its funders should be clear about assignment of responsibility for research translation activities. We agree with Burke and colleagues, who conclude, on the basis of their experiences with the "translational imperative" of the CEER program, that it would be a mistake to require that ELSI research programs demonstrate a direct impact on science or health policy.[63] However, for the ELSI Research Program to have maximum utility, it should facilitate the translation of ELSI scholarship. To that end, we would suggest that it evaluate the appropriateness of requiring academicians to produce policy-relevant grant products, and either discontinue this legacy requirement for grantees (and arrange for this responsibility to belong to well-informed others) or, when it is appropriate, train and firmly support a set of investigators from policy-focused disciplines to create specific products. It could also create pathways for ELSI scholars to engage with a broad range of stakeholders.

It has been asserted (as a criticism) that the ELSI program is principally designed to support the production of academic research.[29] If this is so, what challenges does its potential entanglement with the strategic priorities of the NHGRI pose for the classification of ELSI as a field of intellectual inquiry? Its strategic plans suggest that NHGRI has been supporting basic science research on the human genome for the purpose of its eventual clinical translation and use.[25,58,94] The achievement of this goal will ultimately require the recruitment of thousands of subjects to genomic research, the adoption of genomic technologies in the clinic, and their acceptance by patients. The singular focus suggested by this project may partly explain the gap in the NHGRI ELSI portfolio identified by the ERPEG in 2000, namely, a lack of studies that explored the broader social implications of enhanced knowledge of human genetics and genomics. Work that has no translational potential may not be prioritized for addition to the portfolio. Although out of scope for the present paper, an updated portfolio review may identify evidence that many funded projects are designed to recruit members of the public to the NHGRI project. It may find additional gaps, especially in areas that may be critical of genome science.

There are also questions that may be beyond the scope of NHGRI ELSI Research Program grantees. For example, the suggestion by Fabi and Goldberg that a focus by bioethics funders on "genetics, genomics, neuroethics, and the ethics of other emerging technologies disproportionately harms People of Color."[95] They argued for a more just allocation of research dollars to the field of bioethics because "a narrow focus on emerging technologies, such as genetic and genomic technologies, reflects a priority set that does not always represent the needs of all sectors of society."[95] Internal and external evaluations have suggested that the ELSI Research Program has struggled to achieve demographic diversity in its recruitment of new investigators.[52,57,95] The allocation of research funding to ELSI, especially if the ELSI program continues to struggle in this way, could limit the kinds of research questions that are explored as well as contribute to the existing, unequal allocation of NIH funding to scholars of color.[95–97] Perhaps the outcome here depends upon the results of the NIH anti-racism initiative, UNITE,

announced in March 2021, which aims to reduce barriers to achieving racial equity in the NIH-supported and external scientific workforce (including ELSI).[98] It also depends upon the actions of other institutions in the bioethics ecosystem.[95]

The past three decades of sustained investment by NHGRI have created a substantial body of scholarship and successive cohorts of trained ELSI practitioners. These investments enable emerging ELSI issues to be described with greater specificity and approached with accumulated knowledge and experience. Our experience will be of great value in the coming years as ELSI scholars find themselves engaged in a study of genome science that "has become increasingly woven into the fabric of biomedical research, medical practice, and society."[64] As the reach of genome science stretches beyond its laboratory origins, it is more imperative that ELSI strive to be an independent field of inquiry driven by the needs and concerns of those who are affected by developments in genome science. If NHGRI and genome science are ready to seriously examine the social and environmental conditions that interact with genetic risk in the production of human disease, the urgent need to engage diverse communities, and the varied personal and cultural influences on the interpretation and use of genetic information, it should look to ELSI researchers, who now can productively draw on thirty years of experience, to make meaningful contributions to the analysis of those issues in the fourth decade of ELSI study.

SUPPLEMENTAL INFORMATION

Supplemental information can be found online at https://doi.org/10.1016/j.xgen.2022.100150.

ACKNOWLEDGMENTS

We would like to thank Eric Juengst and the anonymous reviewers for their comments, Joy Boyer for organizational charts and budget data, and Caroline B. Moore for reference formatting and tables. This essay was supported by the National Human Genome Research Institute at the National Institutes of Health under award U24HG010733. The content is solely the responsibility of the authors and does not represent the official views of the National Institutes of Health.

AUTHOR CONTRIBUTIONS

Conceptualization, D.D.D., S.S.-J.L., and M.K.C.; formal analysis, D.D.D.; investigation, D.D.D.; writing – original draft, D.D.D.; writing – review & editing, D.D.D., S.S.-J.L., and M.K.C.; funding acquisition, S.S.-J.L. and M.K.C.; supervision, M.K.C.; visualization, D.D.D.; project administration, D.D.D.

DECLARATION OF INTERESTS

All authors declare salary support from the ELSI Research Program, National Human Genome Research Institute, National Institutes of Health. S.S.-J.L. was a member of the Genomics and Society Working Group from 2016 to 2020 and currently serves on the National Academies of Science, Engineering and Medicine Committee on Use of Race, Ethnicity, and Ancestry as Population Descriptors in Genomics Research.

REFERENCES

1. Morrissey, C., and Walker, R.L. (2012). Funding and forums for ELSI research: who (or what) is setting the agenda? AJOB Prim. Res. *3*, 51–60. https://doi.org/10.1080/21507716.2012.678550.
2. McEwen, J.E., Boyer, J.T., and Sun, K.Y. (2013). Evolving approaches to the ethical management of genomic data. Trends Genet. *29*, 375–382. https://doi.org/10.1016/j.tig.2013.02.001.
3. Zwart, H., Landeweerd, L., and van Rooij, A. (2014). Adapt or perish? Assessing the recent shift in the European research funding arena from 'ELSA' to 'RRI'. Life Sci. Soc. Pol. *10*, 11. https://doi.org/10.1186/s40504-014-0011-x.
4. Zwart, H., and Nelis, A. (2009). What is ELSA genomics? EMBO Rep. *10*, 540–544. https://doi.org/10.1038/embor.2009.115.
5. Kosseim, P., and Chapman, S. (2011). Science and society: some "Made-in-Canada" options for improving integration. Account. Res. *18*, 194–216. https://doi.org/10.1080/08989621.2011.575246.
6. Chadwick, R., and Zwart, H. (2013). From ELSA to responsible research and promisomics. Life Sci. Soc. Pol. *9*, 3. https://doi.org/10.1186/2195-7819-9-3.
7. López, J.J., and Lunau, J. (2012). ELSIfication in Canada: legal modes of reasoning. Sci. Cult. *21*, 77–99. https://doi.org/10.1080/09505431.2011.576240.
8. López, J.J., and Robertson, A. (2007). Ethics or politics?: the emergence of ELSI discourse in Canada. Can. Rev. Sociol. *44*, 201–218. https://doi.org/10.1111/j.1755-618X.2007.tb01134.x.
9. Hartman, A.L., Hechtelt Jonker, A., Parisi, M.A., Julkowska, D., Lockhart, N., and Isasi, R. (2020). Ethical, legal, and social issues (ELSI) in rare diseases: a landscape analysis from funders. Eur. J. Hum. Genet. *28*, 174–181. https://doi.org/10.1038/s41431-019-0513-3.
10. Rip, A. (2016). The clothes of the emperor. An essay on RRI in and around Brussels. J. Responsible Innov. *3*, 290–304. https://doi.org/10.1080/23299460.2016.1255701.
11. Stegmaier, P. (2009). The rock 'n' roll of knowledge co-production. EMBO Rep. *10*, 114–119. https://doi.org/10.1038/embor.2008.253.
12. Hilgartner, S., Prainsack, B., and Hurlbut, J.B. (2016). Ethics as governance in genomics and beyond. In Handbook of Science and Technology Studies, 4th ed., U. Felt, R. Fouché, C.A. Miller, and L. Smith-Doerr, eds. (MIT Press), pp. 823–852.
13. Penders, B., Horstman, K., and Vos, R. (2008). A ferry between cultures: crafting a new profession at the intersection of science and society. EMBO Rep. *9*, 709–713. https://doi.org/10.1038/embor.2008.134.
14. Van Est, R. (2011). The broad challenge of public engagement in science. Sci. Eng. Ethics *17*, 639–648. https://doi.org/10.1007/s11948-011-9296-9.
15. Nydal, R., Myhr, A.I., and Myskja, B.K. (2015). From ethics of restriction to ethics of construction: ELSA research in Norway. Nordic J. Sci. Technol. Stud. *3*, 34–45. https://doi.org/10.5324/njsts.v3i1.2155.
16. Mehlich, J. (2017). "Is, ought, should"—scientists' role in discourse on the ethical and social implications of science and technology. Palgrave Commun. *3*, 17006. https://doi.org/10.1057/palcomms.2017.6.
17. Huijer, M. (2006). Between dreams and reality: the Dutch approach to genomics and society. BioSocieties *1*, 91–95. https://doi.org/10.1017/S1745855205050143.
18. Forsberg, E.M. (2014). Institutionalising ELSA in the moment of breakdown? Life Sci. Soc. Pol. *10*, 1. https://doi.org/10.1186/2195-7819-10-1.
19. Yoon, J.R., Cho, S.K., and Jung, K.W. (2010). The challenges of governing biotechnology in Korea. East Asian Sci. Technol. Soc. Int. J. *4*, 335–348. https://doi.org/10.1215/s12280-010-9131-4.
20. Ramsay, S. (2001). Ethical implications of research on the human genome. Lancet *357*, 535. https://doi.org/10.1016/S0140-6736(05)71689-5.
21. van der Weele, C. (2008). Moral agendas for genomics: how to find the blind spots? Configurations *16*, 117–135. https://doi.org/10.1353/con.0.0042.
22. Cho, M.K. (2012). ELSI research and genetics: a co-evolution. AJOB Prim. Res. *3*, 1–2. https://doi.org/10.1080/21507716.2012.688786.

23. Parker, L.S., Sankar, P.L., Boyer, J., Jean McEwen, J., and Kaufman, D. (2019). Normative and conceptual ELSI research: what it is, and why it's important. Genet. Med. *21*, 505–509. https://doi.org/10.1038/s41436-018-0065-x.

24. Walker, R.L., and Morrissey, C. (2012). Charting ELSI's future course: lessons from the recent past. Genet. Med. *14*, 259–267. https://doi.org/10.1038/gim.2011.60.

25. Collins, F.S., Patrinos, A., Jordan, E., Chakravarti, A., Gesteland, R., and Walters, L. (1998). New goals for the U.S. Human genome project: 1998–2003. Science *282*, 682–689. https://doi.org/10.1126/science.282.5389.682.

26. Annas, G.J. (1989). At law: who's afraid of the human genome? Hastings Cent. Rep. *19*, 19. https://doi.org/10.2307/3562296.

27. Rothenberg, K.H., and Thomson, E.J. (1994). Women and Prenatal Testing: Facing the Challenges of Genetic Technology (The Ohio State University Press).

28. Lombardo, P. (2011). A Century of Eugenics in America: From the Indiana Experiment to the Human Genome Era (Indiana University Press).

29. Hanna, K.E. (1995). The ethical, legal, and social implications program of the National Center for Human Genome Research: a missed opportunity? In Society's Choices: Social and Ethical Decision Making in Biomedicine, R.E. Bulger, E.M. Bobby, and H.V. Fineberg, eds. (National Academy Press), pp. 432–457.

30. Sabatello, M., and Juengst, E. (2019). Genomic essentialism: its provenance and trajectory as an anticipatory ethical concern. Hastings Cent. Rep. *49*, S10–S18. https://doi.org/10.1002/hast.1012.

31. National Research Council (NRC) (1988). Mapping and Sequencing the Human Genome (National Academy Press).

32. Office of Technology Assessment (OTA) (1988). Mapping Our Genes: Genome Projects How Big? How Fast? (U.S. Government Printing Office). OTA-BA-373.

33. Juengst, E.T. (1996). Self-critical federal science? The ethics experiment within the U.S. Human Genome Project. Soc. Philos. Pol. *13*, 63–95. https://doi.org/10.1017/S0265052500003460.

34. Marshall, E. (1996). The genome program's conscience. Science *274*, 488–490. https://doi.org/10.1126/science.274.5287.488.

35. Annas, G.J., and Elias, S. (1992). Gene Mapping: Using Law and Ethics as Guides (Oxford University Press).

36. Cold Spring Harbor Laboratory Library & Archives (2016). The human genome project: an annotated scholarly guide to the project in the United States. http://library.cshl.edu/Guide-to-HGP/Guide.pdf.

37. Watson, J.D. (1990). The human genome project: past, present, and future. Science *248*, 44–49. https://doi.org/10.1126/science.2181665.

38. NHGRI (2012). ELSI planning and evaluation history. https://www.genome.gov/10001754/elsi-planning-and-evaluation-history.

39. Cook-Deegan, R. (1994). The Gene Wars: Science, Politics, and the Human Genome (W. W. Norton & Company).

40. Wolfe, A. (2002). Federal policy making for biotechnology, executive branch, ELSI. In Encyclopedia of Ethical, Legal and Policy Issues in Biotechnology, *1*, T.H. Murray and M.J. Mehlman, eds. (John Wiley & Sons, Inc.), pp. 234–240.

41. Fink, L. (1990). NCHGR bioethics program addresses social and ethical implications of knowledge about human genes. https://repository.library.georgetown.edu/bitstream/handle/10822/556962/52%20Fink%201990%20NCHGR%20Bioethics%20Implications%20Report.pdf?sequence=1.

42. Meslin, E.M., Thomson, E.J., and Boyer, J.T. (1997). The ethical, legal, and social implications research program at the national human genome research institute. Kennedy Inst. Ethics J. *7*, 291–298. https://doi.org/10.1353/ken.1997.0025.

43. House Committee on Government Operations; 102nd Congress (1992). Designing genetic information policy: the need for an independent policy review of the ethical, legal, and social implications of the human genome project. https://babel.hathitrust.org/cgi/pt?id=uc1.31822008224339&view=1up&seq=9.

44. Lehrman, S. (1996). Genome ethics panel comes under the microscope at NIH. Nature *381*, 357. https://doi.org/10.1038/381357a0.

45. Larson, E.J. (1993). Half a tithe for ethics. Natl. Forum *73*, 12–14.

46. Juengst, E.T. (1994). Human genome research and the public interest: progress notes from an American science policy experiment. Am. J. Hum. Genet. *54*, 121–128.

47. Zylke, J.W. (1992). Examining life's (genomic) code means reexamining society's long-held codes. J. Am. Med. Assoc. *267*, 1715. https://doi.org/10.1001/jama.1992.03480130021002.

48. 103rd Congress (1993). National Institutes of Health revitalization act of 1993. https://www.congress.gov/103/bills/s1/BILLS-103s1enr.pdf.

49. Andrews, L.B. (1999). The Clone Age: Adventures in the New World of Reproductive Technology (Henry Holt and Company).

50. Lehrman, S. (1996). Genome ethics chair resigns amid worries over autonomy. Nature *380*, 96. https://doi.org/10.1038/380096a0.

51. Rothstein, M.A., Spence, M.A., Buffler, P.A., Childress, J.F., Epstein, C.J., Hilgartner, S., Knoppers, B.M., Mackta, J., Olson, M.V., Shine, K.I., et al. (1996). Report of the joint NIH/DOE Committee to evaluate the ethical, legal and social implications program of the human genome project. https://www.genome.gov/10000478/nihdoe-elsi-program-evaluation.

52. Walters, L.B., Clayton, E.W., Fisher, N.L., Lerman, C.E., McInerney, J.D., Nebo, W.E., Press, N., Valle, D., Boyer, J.T., Drell, D., et al. (2000). A review and analysis of the ethical, legal, and social implications (ELSI) research programs at the National Institutes of Health and the Department of Energy: final report of the ELSI research planning and evaluation group. https://www.genome.gov/Pages/Research/DER/ELSI/erpeg_report.pdf.

53. NHGRI (2020). NHGRI history and timeline of events. https://www.genome.gov/about-nhgri/Brief-History-Timeline.

54. Annas, G.J., Glantz, L.H., and Roche, P.A. (1995). The genetic privacy act and commentary. https://www.osti.gov/biblio/395609-genetic-privacy-act-commentary.

55. Collins, F.S., Green, E.D., Guttmacher, A.E., and Guyer, M.S. (2003). A vision for the future of genomics research. Nature *422*, 835–847. https://doi.org/10.1038/nature01626.

56. NHGRI (2006). The role of ELSI research & policy activities in the NHGRI plan. https://www.genome.gov/10005516/elsi-role-report.

57. Ethical, Legal, and Social Implications (ELSI) Research Advisors (ERA) (2005). Report to the National Advisory Council for human genome research. https://elsihub.org/sites/default/files/content-files/ERAReportdraft5.pdf.

58. Green, E.D., and Guyer, M.S. (2011). Charting a course for genomic medicine from base pairs to bedside. Nature *470*, 204–213. https://doi.org/10.1038/nature09764.

59. NHGRI (2017). NHGRI reorganized to meet expanding research mission. https://www.genome.gov/27549973/2012-release-nhgri-reorganized-to-meet-expanding-research-mission.

60. Boyer, J.T., Lawrence, B.C., Kaufman, D., Lockhart, N.C., and McEwen, J.E. (2017). ELSI research programme of the NHGRI. In eLS, Angus Clarke, ed. (John Wiley & Sons, Ltd.).

61. McEwen, J.E., Boyer, J.T., Sun, K.Y., Rothenberg, K.H., Lockhart, N.C., and Guyer, M.S. (2014). The ethical, legal, and social implications program of the national human genome research institute: reflections on an ongoing experiment. Annu. Rev. Genom. Hum. Genet. *15*, 481–505. https://doi.org/10.1146/annurev-genom-090413-025327.

62. NHGRI (2021). Ethical, legal and social implications research program. https://www.genome.gov/Funded-Programs-Projects/ELSI-Research-Program-ethical-legal-social-implications#areas.

63. Burke, W., Appelbaum, P., Dame, L., Marshall, P., Press, N., Pyeritz, R., Sharp, R., and Juengst, E. (2015). The translational potential of research

on the ethical, legal, and social implications of genomics. Genet. Med. *17*, 12–20. https://doi.org/10.1038/gim.2014.74.

64. National Institutes of Health (2019). Limited competition centers of excellence in ethical, legal and social implications (ELSI) research (CEER) (RM1 clinical trial optional). https://grants.nih.gov/grants/guide/rfa-files/RFA-HG-19-012.html.

65. Oliver, J.M., and McGuire, A.L. (2011). Exploring the ELSI universe: critical issues in the evolution of human genomic research. Genome Med. *3*. https://doi.org/10.1186/gm254.

66. Kaye, J., Meslin, E.M., Knoppers, B.M., Juengst, E.T., Deschênes, M., Cambon-Thomsen, A., Chalmers, D., De Vries, J., Edwards, K., Hoppe, N., et al. (2012). ELSI 2.0 for genomics and society. Science *336*, 673–674. https://doi.org/10.1126/science.1218015.

67. Bell, J., Ancillotti, M., Coathup, V., Coy, S., Rigter, T., Tatum, T., Grewal, J., Akcesme, F.B., Brkić, J., Causevic-Ramosevac, A., et al. (2016). Challenges and opportunities for ELSI early career researchers. BMC Med. Ethics *17*, 1–6. https://doi.org/10.1186/s12910-016-0121-5.

68. Clayton, E.W., Smith, M., Fullerton, S.M., Burke, W., McCarty, C.A., Koenig, B.A., McGuire, A.L., Beskow, L.M., Dressler, L., Lemke, A.A., et al. (2010). Confronting real time ethical, legal, and social issues in the Electronic Medical Records and Genomics (eMERGE) Consortium. Genet. Med. *12*, 616–620. https://doi.org/10.1097/GIM.0b013e3181efdbd0.

69. McGuire, A.L., Colgrove, J., Whitney, S.N., Diaz, C.M., Bustillos, D., and Versalovic, J. (2008). Ethical, legal, and social considerations in conducting the Human Microbiome Project. Genome Res. *18*, 1861–1864. https://doi.org/10.1101/gr.081653.108.

70. Wright, G.E.B., Koornhof, P.G.J., Adeyemo, A.A., and Tiffin, N. (2013). Ethical and legal implications of whole genome and whole exome sequencing in African populations. BMC Med. Ethics *14*, 1–15. https://doi.org/10.1186/1472-6939-14-21.

71. Balmer, A.S., Calvert, J., Marris, C., Molyneux-Hodgson, S., Frow, E., Kearnes, M., Bulpin, K., Schyfter, P., Mackenzie, A., and Martin, P. (2015). Taking roles in interdisciplinary collaborations: reflections on working in post-ELSI spaces in the UK synthetic biology community. Sci. Technol. Stud. *28*, 25. https://doi.org/10.23987/sts.55340.

72. Joly, Y., So, D., Saulnier, K., and Dyke, S.O.M. (2016). Epigenetics ELSI: darker than you think? Trends Genet. *32*, 591–592. https://doi.org/10.1016/j.tig.2016.07.001.

73. Annas, G.J. (2003). Adventures with Lori Andrews. J. Contemp. Health Law Pol. *19*.

74. Conley, J.M., Prince, A.E.R., Davis, A.M., Cadigan, J., and Lazaro-Munoz, G. (2020). Is real-time ELSI realistic? AJOB Empir. Bioeth. *11*, 134–144. https://doi.org/10.1080/23294515.2020.1722289.

75. Seltzer, D., Zoloth, L., Traina, C.L., and Kiesling, L. (2011). Paved with good intentions: rethinking the ethics of ELSI research. J. Res. Adm. *42*, 15–24.

76. Yesley, M.S. (1998). Protecting genetic difference. Berk. Technol. Law J. *13*, 653–665. https://doi.org/10.15779/Z38KX1J.

77. Yesley, M.S. (2008). What's ELSI got to do with it? Bioethics and the human genome project. New Genet. Soc. *27*, 1–6. https://doi.org/10.1080/14636770701843527.

78. Caulfield, T., Chandrasekharan, S., Joly, Y., and Cook-Deegan, R. (2013). Harm, hype and evidence: ELSI research and policy guidance. Genome Med. *5*, 21. https://doi.org/10.1186/gm425.

79. Caulfield, T. (2016). Ethics hype? Hastings Cent. Rep. *46*, 13–16. https://doi.org/10.1002/hast.612.

80. Fisher, E. (2005). Lessons learned from the ethical, legal and social implications program (ELSI): planning societal implications research for the national nanotechnology program. Technol. Soc. *27*, 321–328. https://doi.org/10.1016/j.techsoc.2005.04.006.

81. Annas, G.J., Glantz, L.H., and Roche, P.A. (1995). Drafting the genetic privacy act: science, policy and practical considerations. J. Law Med. Ethics *23*, 360–366. https://doi.org/10.1111/j.1748-720x.1995.tb01378.x.

82. DeWeese, P.J. (2002). The ELSI research program and genetic nondiscrimination legislation: a study in public policy. https://vtechworks.lib.vt.edu/handle/10919/42773.

83. 110th Congress (2008). Genetic information nondiscrimination act of 2008. PL 110-233. https://www.govinfo.gov/content/pkg/PLAW-110publ233/pdf/PLAW-110publ233.pdf.

84. UN Educational, Scientific and Cultural Organization (UNESCO) (1997). Universal declaration on the human genome and human rights. http://portal.unesco.org/en/ev.php-URL_ID=13177&URL_DO=DO_TOPIC&URL_SECTION=201.html.

85. Collins, F.S. (2004). What we do and don't know about 'race', 'ethnicity', genetics and health at the dawn of the genome era. Nat. Genet. *36*, S13–S15. https://doi.org/10.1038/ng1436.

86. Executive Office of the President (2000). To prohibit discrimination in federal employment based on genetic information. https://www.federalregister.gov/documents/2000/02/10/00-3331/to-prohibit-discrimination-in-federal-employment-based-on-genetic-information.

87. Shen, H. (2013). US brain project puts focus on ethics. Nature *500*, 261–262. https://doi.org/10.1038/500261a.

88. Skirton, H., Goldsmith, L., Jackson, L., O'Connor, A., and O'Connor, A. (2012). Direct to consumer genetic testing: a systematic review of position statements, policies and recommendations. Clin. Genet. *82*, 210–218. https://doi.org/10.1111/j.1399-0004.2012.01863.x.

89. Wolf, S.M., Lawrenz, F.P., Nelson, C.A., Kahn, J.P., Cho, M.K., Clayton, E.W., Fletcher, J.G., Georgieff, M.K., Hammerschmidt, D., Hudson, K., et al. (2008). Managing incidental findings in human subjects research: analysis and recommendations. J. Law Med. Ethics *36*, 219–248. https://doi.org/10.1111/j.1748-720X.2008.00266.x.

90. Yudell, M., Roberts, D., DeSalle, R., and Tishkoff, S. (2016). Taking race out of human genetics. Science *351*, 564–565. https://doi.org/10.1126/science.aac4951.

91. Yudell, M., Roberts, D., DeSalle, R., and Tishkoff, S. (2020). NIH must confront the use of race in science. Science *369*, 1313–1314. https://doi.org/10.1126/science.abd4842.

92. Department of Health and Human Services; Office of the Secretary (2002). Establishment of the secretary's advisory committee on genetics, health, and society. https://www.federalregister.gov/documents/2002/10/23/02-27030/establishment-of-the-secretarys-advisory-committee-on-genetics-health-and-society.

93. Juengst, E.T. (2021). Anticipating the ethical, legal, and social implications of human genome research: an ongoing experiment. Am. J. Med. Genet. *185*, 3369–3376. https://doi.org/10.1002/ajmg.a.62405.

94. Green, E.D., Gunter, C., Biesecker, L.G., Di Francesco, V., Easter, C.L., Feingold, E.A., Felsenfeld, A.L., Kaufman, D.J., Ostrander, E.A., Pavan, W.J., et al. (2020). Strategic vision for improving human health at the forefront of genomics. Nature *586*, 683–692. https://doi.org/10.1038/s41586-020-2817-4.

95. Fabi, R., and Goldberg, D.S. (2020). Bioethics, (funding) priorities, and the perpetuation of injustice. AJOB *22*, 6–13. https://doi.org/10.1080/15265161.2020.1867934.

96. Hoppe, T.A., Litovitz, A., Willis, K.A., Meseroll, R.A., Perkins, M.J., Hutchins, B.I., Davis, A.F., Lauer, M.S., Valantine, H.A., Anderson, J.M., and Santangelo, G.M. (2019). Topic choice contributes to the lower rate of NIH awards to African-American/black scientists. Sci. Adv. *5*, eaaw7238. https://doi.org/10.1126/sciadv.aaw7238.

97. Taffe, M.A., and Gilpin, N.W. (2021). Racial inequity in grant funding from the US National Institutes of Health. Elife *10*, e65697. https://doi.org/10.7554/elife.65697.

98. National Institutes of Health (2021). UNITE. https://www.nih.gov/ending-structural-racism/unite.

Cell Genomics, Volume *2*

Supplemental information

Three decades of ethical, legal, and social implications research: Looking back to chart a path forward

Deanne Dunbar Dolan, Sandra Soo-Jin Lee, and Mildred K. Cho

Title

Author list: Deanne Dunbar Dolan, Sandra Soo-Jin Lee, Mildred K. Cho

Summary

Initial submission: Received : **Mar 25, 2021**

Scientific editors: Orli Bahcall, Rosalind Mott, Laura M. Zahn

First round of review: Number of reviewers: 2
Revision invited : Sep 9 2021

Revision received : Feb 23 2022

Second round of review: Number of reviewers: 1
Accepted :

Data freely available: N/A

Code freely available: N/A

This transparent peer review record is not systematically proofread, type-set, or edited. Special characters, formatting, and equations may fail to render properly. Standard procedural text within the editor's letters has been deleted for the sake of brevity, but all official correspondence specific to the manuscript has been preserved.

Referees' reports, first round of review

Reviewer 1:

Tremendously difficult to summarize 30 years of ELSI research and politics without taking "sides." Overall, I thought you did a reasonable job, but I would push you further to look more deeply at three issues: (1) the original agenda from the perspective of James Watson and also the ELSI working group; (2) the total control taken by Francis Collins of the ELSI Working Group after Lori Andrews resigned as its chairperson (recounted in her book, The Clone Age), and the simultaneous movement of "public policy" away from suggesting specific laws and regulations, especially in the arena of genetic privacy; and (3) the intellectual and practical relationship between ELSI researchers and the National Academy of Medicine reports on genetics (including gene editing).

Some specifics: (1) Of some historical and intellectual interest, the first extramural workshop funded by NIH was held in Bethesda in January 1991. Its purpose was to develop a prioritized social policy research agenda for the Human Genome Project. The four areas the group suggested, in rank order, were: 1. When and how should new genetic tests be introduced into medical practice?; How can confidentiality and privacy of an individual's genetic information be preserved?; How can genetic discrimination by employers and insurance companies be prevented?; and 4. How might the Human Genome Project affect our concepts of 'disease,' 'normalcy', and 'humanness.'? Annas, GJ & Elias S, eds, Gene Mapping: Using Law & Ethics as Guides, Oxford U. Press, 1992. Although not stated in these exact terms, these four items remain on the top of any ELSI research priorities to this day. (2) The resignation of Lori Andrews as chair of the ELSI working group (which you note) is of much more influence over the ELSI agenda than you give it credit for. It marked the end of any independence of the ELSI research agenda, removing the Working Group's independence (such as it was), and putting complete control in the hands of Francis Collins (who had by then replaced James Watson). In retrospect, Prof. Andrews suggests that ELSI may not have been seen as all that important by Watson either. In her words, "At a genetics policy meeting I learned why James Watson had formed the ELSI Working Group. Watson implied that the ELSI Working Group had been created not to set ethical standards but to let the science proceed unimpeded. 'I wanted a group that would talk and talk and never get anything done,' Watson said, 'and if they did do something, I wanted them to get it wrong.' 'I wanted as its head Shirley Temple Black." Lori Andrews, The Clone Age, Henry Holt, 1999 at 206. (3) The question of whether ELSI should get directly into making policy is a good one, but one cut short by the resignation of Lori Andrews (over a related issue). With funding from the DOE's ELSI project, researchers responded to the request to draft a federal genetic privacy act. This project was completed in February 1995 (Annas, Glantz & Roche, The Genetic Privacy Act and Commentary (1995). The act was presented to a meeting of the ELSI Working Group in 1994, and got unanimous "approval" at the meeting. Thereafter, researchers (especially, but not only, pathologists) began a campaign against the draft (worried especially that giving individuals an ownership interest in their DNA could make genetic research harder to do), which in the end persuaded Francis Collins not to support this bill. The experience, coupled with the resignation of Lori Andrews at about

the same time, seemed to make ELSI far less likely to engage in legislative drafting in the realm of genomics. (There is some irony that concerns about genetic privacy have more recently taken a front seat in the "All of us" project. As you put it (correctly): "The 1996 ELSI Evaluation Committee recommended the exclusion of direct policy formulation from the remit of the ELSI program." This helps explain why.

I would suggest that you move the material on "Global ELSI Initiatives" to an appendix because as a list without any attempt at synthesis or analysis it doesn't add enough to your paper to warrant the space it is given.

Finally, I'm not convinced that your survey of the ELSI literature leads to your final conclusion, and I ask that you reconsider it.

Reviewer 2:
This is an excellent review of the history of the ELSI program, and builds on the previous efforts (McEwen, Juengst, etc.). It is clearly written and timely. It should be published, and can be an input to NHGRI (and NIH more widely) thinking through where it wants to go with the ELSI research program, now that the "pause" in the CEER program is coming to an end, and NIH's budget it slated for renewed growth.

This piece is stronger on descriptive history than analysis of the lessons learned. I have very few comments about the history, except to suggest that they add a citation to Lori Andrews's chapter from her book "The Clone Age" that focuses on the aspects of the ELSI program as PR front for genomics science, a "shield" to insulate the science from criticism so the science can proceed apace. Jim Watson gave her an unguarded interview, and this was indeed one of his intents, but he was also sincerely interested in "this is not just for scientists to decide," and he was no doubt showcasing the provocateur self when he talked about the purpose of the program. But it's probably the best and sharpest such critique of the program. Lori was still smarting from resigning as ELSI Working Group chair five years earlier, and the chapter is not fully balanced. I don't have access to my copy, but there is a chapter that focuses on this issue that is sharper than the Juengst and other critiques cited.

The article is well worth publishing for the update of the history, and also adding some details to that history, no doubt benefiting from Chris Donahue's historical work at NHGRI.

But its main use will be to guide decisions in the future, and as an analytical piece about "lessons learned" it could be strengthened to good effect. The goals of the program are diverse. Most of the items on the 1989 initial Working Group list and the revamped goals after Francis came to NCHGR are oriented to helping identify issues (early warning and the initial conferences to orient the field), and then to study issues that were cropping up in the science and application (the early work on genetic testing for CF, breast cancer, Alzheimer's, Huntington's, etc.). Some projects have been deep "embedded" projects (EMERGE, CSER, ClinGen have ELSI components, for example), and this "embed" notion has spread to some extent to All of Us and BRAIN Initiatives. But it's striking the degree to which it is *not* been adopted by other institutes even at NIH, and there's little discussion of how and why the DOE program dissipated, despite the very strong Congressional signal that DOE needed to have an ELSI component to its science. And where the "parallel research program" for ELSI has been adopted (e.g.,

the US nanotech, or the Canadian GenomeCanada GE3LS), sometimes each major project is expected to have an ELSI component, and sometimes ELSI research is separately and independently funded. What are the strengths and weaknesses of the "embed" versus "independent" modes? This is crucial to NHGRI's internal debate about the role of CEER centers, and this article is an opportunity to signal the community how NHGRI is thinking about that central question.

As one niggle with the history. The Walters and Rothstein evaluations of the program were partly a reaction to Nancy Wexler, Lori Andrews and Troy Duster succession of working group leadership, but those resignations were symptoms as much as causes. The underlying tension was whether Francis and Kathy Hudson would control the agenda, or an external group. And the role of the working group in formulating policy was hotly contested, and indeed the main bone of contention. The evaluations did indeed caution against formulating policy within an NIH advisory committee (which is what the working group was—or should have been explicitly framed to be, a FACA committee). But that is a clear divergence from the expectations in Congress, and the initial goals (see #4 of the 1989/1990 "develop policy options" that Leslie Fink included in her summary). The real frustration of folks like Kathy and Francis trying to get things done in a highly political environment and the independence of an advisory committee and a research program that was not tightly harnessed to NCHGR/NHGRI policy priorities but left to investigators and study sections does not really come through. And the decision to remove the "policy options" function was a judgement call, and one that was (and should have been) hotly contested. Those evaluation reports were crucial in shifting the main power to set the ELSI research agenda to internal NHGRI documents and staff, and to reduce the independence and power of the working group as a national bioethics committee (and it was serving both functions, to some degree—witness the ELSI working group task forces on insurance and on genetic testing). One interpretation is that the evaluations were intended to rein in the ELSI Working Group from those policy-oriented activities, a goal that was largely accomplished, as the result is an group of ELSI Research Advisors who plug into the NIH-standard advisory council. That group has much lower profile, focused on the ELSI research agenda, and shed the "national bioethics forum" aspirations. That certainly simplified NHGRI politics by reducing the political salience of the ELSI working group? But was what was good for NHGRI management good for the country? I don't know the answer to that, and probably no one does, but it was a fateful choice that should be explicitly addressed. The framing of the tasks for those evaluation committees was crucially determined by Kathy and Francis. That history is pretty well known to those who lived through it, and the current account seems bowdlerized.

The ELSI program is housed in a research institute that fosters some very basic research. Some of the ELSI grants were intended to roughly parallel this model in supporting purely intellectual contributions to advance the field—that is, unfettered investigator-initiated research. How much? How have those fared. The list of problems that ELSI research has helped to address is relevant to the "problem solving" and "early warning" goals, which stem from a more practical rationale for the research (more "applied" in Vannevar Bush's terms). How much of the ELSI portfolio is devoted to that?

In general, the piece would be greatly strengthened if it included some evaluation, specified the different goals explicitly, included some funding and portfolio-analysis data that is expected of any NIH program evaluation, and some "strengths and weaknesses" assessment of how the program has achieved (or not) the goals. The history is there, now flesh it out with some funding data, e.g., fraction of

ELSI relative to NHGRI budget overall, fraction devoted to CEERs, fraction devoted to "intellectual advancement" versus "problem solving" versus "field support (e.g., the CERA and T32s) versus "embedded ELSI" in major tech initiatives. These overlap, so the analysis cannot be simple and completely clean, but these are the real issues facing the field, and these problems of classification and analysis plague the scientific programs at NIH just as much as ELSI. And yet NIH has a history of program reviews that matter.

The main value of this article will be to review and update the history of the ELSI program. But one important purpose of that history is to inform the choices facing NHGRI for the program's future, and to address why the model has remained largely restricted to NHGRI. Three decades ago, OTA and the Institute of Medicine recommended that the ADAMHA institutes have ELSI programs. They never really developed them. Why? Imagine, if you will, if NIAID had an ELSI component (beyond strong ties to the Clinical Center bioethics program) to complement its vaccine-development effort, and how it might have anticipated the issues that have proven to be major obstacles to pandemic management (and those obstacles have largely been social, political, and economic, not just science and technology). Or how NIH is grappling with AI and infotech, with the usual highly decentralized institute-and-Center responses that are somewhat coordinated, but largely bereft of the kind of social science research that could have been going on for the past decade if NIH had a capacity for exploring ELSI of biomedical research generally, analogous to NHGRI's capacity for ELSI of genomics.

This critique may seem harsh and add too much work, but the NHGRI ELSI program really is the largest and longest-standing such program in the world, and now decisions are being made about its future configuration. This article is an opportunity to do the hard work of thinking through what that might look like, based on historical *assessment* not just historical description (which this piece does a really nice job of). I would encourage the author to do some of that hard work. Perhaps not in this piece (as it's already fairly long), but perhaps by alluding to (and pledging to write) a future analysis. But if that is the solution chosen, the current history is still incomplete with out the budget data and specification of goals and approaches noted above. A paragraph that lays out the "portfolio" of ELSI projects with different goals, and the budget breakdowns used to guide current NHGRI decisions should be laid out. And one result might be to refine those categories and clarify the disparate goals and adjust the metrics to accommodate the diverse goals of different projects. (As one example, the "innovation" criterion for grants is highly relevant for projects funded to advance ELSI as a field; but it is actually counterproductive for most "embedded" projects where the purpose is to help channel evidence about what's known to project leaders and anticipate issues using standard methods that are tried-and-true, because there the purpose of ELSI is to improve the science, not advance ELSI as a field—except as "embeddedness" can itself be subject to some innovation, but it really is not the main purpose.)

Finally, there's an intellectual incoherence embedded in the final few pages. Sometimes ELSI is described as a field. It's clearly not a discipline, but rather something else. And yet the CEERs once had training components and there are ELSI T32 training programs. By those criteria, it is a "field" in the sense of having a community of folks who read one anothers' work and perform Merton's CUDOS functions for that community. But it's not a field in some other senses, as it is sometimes helping solve problems, sometimes forecasting, and sometimes it really is trying to advance intellectual contributions to existing

fields, disciplines, or professions (e.g., clinical practice, patent law, philosophy of biology, moral philosophy). Is it a function (or functions) or a field? Or both?

Authors' response to the first round of review

Dear Dr. Bahcall,

Thank you for a thorough peer-review process and your editorial comments on our March 2021 submission, A history and actionable priorities for the fourth decade of ELSI research. We have substantially revised the work to provide a more balanced account of the ELSI Research Program at the National Human Genome Research Insititute (NHGRI). Specifically, the following reviewer and journal concerns are fully addressed in the revised manuscript:

1. We clarified our claim that ELSI research is a field of study.
2. We described the 1991 workshop that Reviewer 1 felt identified 4 areas of social policy for the HGP and remain the ELSI research priorities today.
3. We moved lists to boxes or tables and have added a table that details the portion of the NHGRI extramural research budget allocated to ELSI over the past 4 years.
4. In order to better characterize the strengths and weaknesses of the ELSI Research Program, we discuss published evaluations by science policy or ethics scholars as well as in-depth, formal evaluations by advisory panels appointed by NHGRI (e.g., Hanna, 1995; Juengst, 1996; Rothstein et al., 1996; Walters et al., 2000; ELSI Research Advisors and ELSI Policy and Planning Group, 2003; and ELSI Research Advisors (ERA), 2005).
5. Given the prominence of investigator-initiated research in the NHGRI ELSI Program portfolio, the content of which is informed both by study sections (peer review) and Council priorities, we were hesitant to follow Lori Anderson's lead and suggest that the NHGRI Director can exercise total control over the ELSI research agenda. We also felt the suggestion by Reviewer 2 that the 1996 external evaluation of the ELSI Working Group and the extramural grants programs at the NIH and the DOE ordered by Collins and Patrinos marked the end of independence for the ELSI research agenda ("Those evaluation reports were crucial in shifing the main power to set the ELSI research agenda to internal NHGRI documents and staff, and to reduce the independence and power of the working group as a national bioethics committee") was somewhat mitigated by the subsequent formation of two national committees focused on the examination of ELSI issues. Nonetheless, we acknowledge this as a possible interpretation of the program history in the new conclusion.
6. The new conclusion provides an interpretation of the program history and recommendations to NHGRI on the future direction of the funding program and the field of study it supports.

Although our revision was substantial, a few remaining issues deserve comment here:

Response to Reviewers

1. We did not include funding or portfolio analysis data for each Center for Excellence in ELSI Research (CEER) because we felt a detailed analysis of CEER budgets, which are only a subset of the overall NIH budget for ELSI research, would be both outside of the current scope and unlikely to contribute meaningfully to the paper.

2. We did not find a published rationale for the sunset of the NHGRI CEER program or the Department of Energy (DOE) ELSI Branch. In the later case, we suspect (but were unable to document) the extramural ELSI funding program run by the DOE was phased out in 2003 with the completion of the HGP. However, the revised paper mentions the CEER sunset, includes detail about the funding differential between the NIH (2/3) and the DOE (1/3) in the HGP, provides a reference for the DOE rationale for joining the HGP (interest in energy-related damage to the human genome), and explains why the DOE funded its ELSI program.
3. We have updated the manuscript to include a breakdown of the NHGRI budget that illuminates the portion spent on investigator-initiated research vs. other areas (training, embedded ELSI, etc). However, the budget data we obtained from NHGRI program staff do not offer the granularity that would be required to divide these expenditures into “purely intellectual” and “applied” categories, as suggested by Reviewer 2. A future analysis that sought to classify individual grants into these categories could be conducted, but we felt the distinction may be too binary and the analysis potentially unproductive.
4. Although we tend to agree with the suggestion by Reviewer 1 that both Andrew’s resignation and a campaign by researchers that persuaded Francis Collins not to support the genetic privacy legislation drafted by George Annas and others with DOE ELSI funding, explains the shifting definition of a “public policy” role for ELSI away from specific laws and regulations, we did not find published support for this idea. Instead, we describe formal, internal evaluations by panels of experts that recommend the relocation of the ELSI Working Group’s policy formulation function to other NIH policy offices. Although we were unable to document the Reviewer’s perspective, we acknowledge this possible interpretation of the program history and make our own argument about the optimal location of science policymaking in the new conclusion.
5. We did not take up the discussion, as suggested by Reviewer 2, of the implications of “embedded” ELSI for the intellectual independence of the field of study. This issue has been discussed in detail elsewhere, e.g., Conley et al., 2020; Joly et al., 2016; Balmer et al., 2015; and Seltzer et al., 2011). Further, at only 3% of the ELSI Research Program budget in 2020, we did not feel that this discussion was especially timely.
6. Reviewer 2 asks that we address why, in their view, the ELSI model has remained largely restricted to NHGRI. While we would love to be in a position to take on this question, we expect that, given the many actors involved (including the U.S. Congress) we would likely find many answers and perhaps none that are directly or definitively motivated by qualities, success, or failures of the ELSI Research Program. As we explain in the manuscript, the ELSI extamural grants programs were the product of a broad set of cultural currents that were unique to the late 1980s. Further, the Reviewer’s contention contradicts our presentation in Table 2 which depicts the broad global adoption of funding set-asides dedicated to ELSI research within national scientific initiatives.

Despite the above limitations and its unusual length compared to a typical Perspectives manuscript, we truly hope the revised manuscript is satisfactory to the reviewers and the journal. Thank you for your further consideration of this work.

Best wishes,

Deanne Dolan, PhD
Stanford University

Referees' report, second round of review

Reviewer 1:
The subject matter is worth the research effort, and your changes were on the whole responsive to the reviewer comments. A few things I would ask you to consider (or re-consider).

1. Although you do cite her main book on the subject, you treat Lori Andrews, a central participant in the debate over governance of the ELSI program as someone you don't seem to trust as an honest reporter. I don't usually suggest that authors be more careful of "tone" but you should in regards to Andrews at least (see, especially where you introduce Andrews as simply "a lawyer" [rather than as a law professor and legal scholar; see eg., Jorge Contreras, The Genome Defense: Inside the Epic Legal Battle to Determine who Owns Your DNA (2021) which recounts the central role Prof. Andrews had in the litigation that eventually led to the Supreme Court decision on gene patenting]; and then when you cite Andrews for important detailed you use the phrase "Andrews claims" (twice on p. 6 alone), and also the phrase "according to Andrews" None of the material attributed to her has been questioned--and if you do not believe her you should say why, not engage in what looks like marginalizing her conclusions based on living through the events. [likewise, at p. 10, "according" to Andrews ...Watson "allegedly"--unlike most of Watson's racist and sexist remarks, this one was at a public event and witnessed by many others, and never denied by Watson. at the least, the "allegedly" should be removed.

2. The proper citation of the "Genetic Privacy Act" (referenced at p. 8) is George J. Annas, Leonard H. Glantz & Patricia A. Roche, The Genetic Privacy Act and Commentary (1995). (easily accessible on the net) The "draft" of this report was presented to the ELSI Working Group at an in person meeting (by Annas) in 1994 and endorsed by the group unanimously, including Francis Collins (who later changed his mind). It may (or may not) be worth noting that virtually all of the consent and privacy provisions of the Genetic Privacy Act were adopted by the "All of Us" project.

3. p. 12, describing the post-Andrews ELSI program as "a public relations stunt" is probably too strong, but there is no doubt that Collins saw genome-related policy making potentially moving out of his office and he moved to contain and control it.(pretty standard Washington behavior)

4. on p. 13: the National Academy of Sciences doesn't really work the way you describe it.

5. Good points (I think) on pages 14 and 15.

6. Its a bit concerning that the only person you acknowledge personally is Eric Juengst who, of course, has his own view (and, for example, who co-authored the foreword to the Gene Mapping book [the source of the research priorities now summarized in box 3] with James Watson.

7. I know its arguably beyond the scope of your paper, but I (and I'm sure many readers) would be very interested in your views on the efforts DARPA has put into developing its own ELSI program (especially in it unclassified Safe Genes project) which, on the surface at least, is homage to the ELSI model.

Authors' response to the second round of review

Dear Dr. Mott,

Thank you for your work as handling editor for our paper. We very much appreciate your support for the expanded Perspective word limit, extra figures/boxes, and additional references. We have reduced the manuscript length from 12,000 to 10,479 words and removed the STAR Methods section, which is not required for the Perspective format. Please find our point-by-point response to the reviewer comments below.

Reviewers' Comments:

Reviewer #1: Subject matter is worth the research effort, and your changes were on the whole responsive to the reviewer comments. A few things I would ask you to consider (or re-consider).

1. Although you do cite her main book on the subject, you treat Lori Andrews, a central participant in the debate over governance of the ELSI program as someone you don't seem to trust as an honest reporter. I don't usually suggest that authors be more careful of "tone" but you should in regards to Andrews at least (see, especially where you introduce Andrews as simply "a lawyer" [rather than as a law professor and legal scholar; see eg., Jorge Contreras, The Genome Defense: Inside the Epic Legal Battle to Determine who Owns Your DNA (2021) which recounts the central role Prof. Andrews had in the litigation that eventually led to the Supreme Court decision on gene patenting]; and then when you cite Andrews for important detailed you use the phrase "Andrews claims" (twice on p. 6 alone), and also the phrase "according to Andrews" None of the material attributed to her has been questioned--and if you do not believe her you should say why, not engage in what looks like marginalizing her conclusions based on living through the events. [likewise, at p. 10, "according" to Andrews ...Watson "allegedly"--unlike most of Watson's racist and sexist remarks, this one was at a public event and witnessed by many others, and never denied by Watson. at the least, the "allegedly" should be removed.

We thank the Reviewer for these comments and have made the suggested corrections.

Our intention was never to disrespect Lori Andrews or her contributions. In round 1, Reviewer #2 cautioned that the relevant chapter in the Clone Age was not fully balanced. Given this, we searched for corroborating accounts of these events in the literature and did not find any. As the Reviewer notes, we also did not find any denial of this account by Watson.

2. The proper citation of the "Genetic Privacy Act" (referenced at p. 8) is George J. Annas, Leonard H. Glantz & Patricia A. Roche, The Genetic Privacy Act and Response to Reviewers Commentary (1995). (easily accessible on the net) The "draft" of this report was presented to the ELSI Working Group at an in person meeting (by Annas) in 1994 and endorsed by the group unanimously, including Francis Collins (who later changed his mind). It may (or may not) be worth noting that virtually all of the consent and privacy provisions of the Genetic Privacy Act were adopted by the "All of Us" project.

We have made this correction. Given we are well over the journal word count, we will thank the reviewer for their insights about the All of Us project and save these details for another paper.

3. p. 12, describing the post-Andrews ELSI program as "a public relations stunt" is probably too strong, but there is no doubt that Collins saw genome-related policy making potentially moving out of his office and he moved to contain and control it.(pretty standard Washington behavior)

We have removed this language.

4. on p. 13: the National Academy of Sciences doesn't really work the way you describe it.

We reviewed our understanding of the cited paper and removed our mention of the NAS after determining that it was not a relevant detail.

5. Good points (I think) on pages 14 and 15.

We thank the reviewer for this comment.

6. Its a bit concerning that the only person you acknowledge personally is Eric Juengst who, of course, has his own view (and, for example, who co-authored the foreword to the Gene Mapping book [the source of the research priorities now summarized in box 3] with James Watson.

We acknowledge Eric Juengst because he read and commented on an earlier version of the manuscript. The reviewer is correct, Juengst co-authored the foreword of the 1992 book, Gene Mapping: Using Law & Ethics as Guides. We suspect this is because, in 1992, James Watson was the Director of the National Center for Human Genome Research (NCHGR) at the NIH and Eric Juengst was the head of the NCHGR ELSI Branch. In round 1, Reviewer #1 asked us to include the social policy research agenda that appeared in Gene Mapping and we did so. We are unaware of any relationship, past or present, between Juengst and Watson that would create a conflict of interest for us.

7. I know its arguably beyond the scope of your paper, but I (and I'm sure many readers) would be very interested in your views on the efforts DARPA has put into developing its own ELSI program (especially in it unclassified Safe Genes project) which, on the surface at least, is homage to the ELSI model.

We thank the reviewer for this comment and agree that this is beyond the scope of the present manuscript.

We hope these responses and the revised manuscript are satisfactory to the reviewers and the journal. Thank you for your further consideration of this work.

Best wishes,

Deanne Dolan, PhD

Stanford University
